# Association of Proximal Tubule Secretion With Hyperkalemia Risk, Treatment Response, and Outcomes in Heart Failure With Preserved Ejection Fraction

**DOI:** 10.1016/j.xkme.2026.101428

**Published:** 2026-06-06

**Authors:** Nicholas Wettersten, Ronit Katz, Pranav S. Garimella, Alexander L. Bullen, Simon B. Ascher, Jesse C. Seegmiller, Yu Horiuchi, Yoshimitsu Takaoka, Joachim H. Ix

**Affiliations:** 1 Cardiology Section, Veterans Affairs San Diego Healthcare System, San Diego, CA; 2 Division of Cardiology, Department of Medicine, University of California San Diego, La Jolla, CA; 3 Division of Obstetrics and Gynecology, Department of Medicine, University of Washington, Seattle, WA; 4 Division of Nephrology-Hypertension, Department of Medicine, University of California San Diego, La Jolla, CA; 5 Nephrology Section, Veterans Affairs San Diego Healthcare System, San Diego, CA; 6 Department of Internal Medicine, University of California Davis, Sacramento, CA; 7 Kidney Health Research Collaborative, Department of Medicine, VA San Francisco Health Care System, University of California, San Francisco, CA; 8 Department of Laboratory Medicine and Pathology, University of Minnesota, Minneapolis, MN; 9 Division of Cardiology, Mitsui Memorial Hospital, Tokyo, Japan

**Keywords:** kidney tubular secretion capacity, heart failure, hyperkalemia, major adverse cardiovascular events, quality of life

## Abstract

**Rationale & Objective:**

Endogenous biomarkers reflecting impaired kidney tubular secretion are associated with risk of hyperkalemia in patients with hypertension and chronic kidney disease. Whether these biomarkers are associated with risk of hyperkalemia, therapeutic response, and adverse cardiovascular and kidney events in heart failure (HF) patients receiving mineralocorticoid receptor antagonists is unknown.

**Study Design:**

An observational cohort study.

**Setting & Participants:**

Individuals with HF with preserved ejection fraction (HFpEF) enrolled in the TOPCAT study.

**Predictors:**

Summary secretion score incorporating urine-to-plasma ratios of 9 secretion markers measured in paired samples at baseline.

**Outcomes:**

Change in serum potassium during follow-up, measures of treatment response assessed by change in Kansas City Cardiomyopathy Questionnaire (KCCQ) score and N-terminal pro-B-type natriuretic peptide over 12 months, and adverse cardiovascular and kidney events.

**Analytic Approach:**

Linear mixed-effects models, Cox proportional hazards models, and linear regression adjusting for risk factors, estimated glomerular filtration rate, and urine albumin-to-creatinine ratio.

**Results:**

Among 372 TOPCAT participants, 179 (48%) were randomized to spironolactone, 45% were women, the mean age was 70 ± 10 years and estimated glomerular filtration rate was 66 ± 18 mL/min/1.73 m^2^. In adjusted models, higher secretion score was not associated with changes in serum potassium, change in KCCQ ≥5 points, change in N-terminal pro-B-type natriuretic peptide, adverse cardiovascular or kidney events. In adjusted models, a higher secretion score was associated with lower KCCQ at 12 months (change −3.5, 95% CI, −6.6 to −0.4) in the spironolactone arm, but not in the placebo arm.

**Limitations:**

Low number of events, confounding from Eastern Europe participants who may have not had HFpEF or taken prescribed therapies.

**Conclusions:**

Biomarkers of tubular secretion are not associated with risk of hyperkalemia, treatment response, or cardiovascular and kidney events in individuals with HFpEF.

## Plain-language Summary

People with chronic kidney disease and hypertension with reduced proximal tubule secretion capacity have a greater risk of hyperkalemia. Whether similar associations are seen in individuals with heart failure is unknown. We studied 372 participants with heart failure with preserved ejection fraction enrolled in the Treatment of Preserved Cardiac Function Heart Failure with an Aldosterone Antagonist study. Using blood and urine specimens collected at the time of enrollment, we measured nine biomarkers of proximal tubule secretion capacity and combined them into a composite secretion score. We then evaluated whether this score was associated with potassium changes, response to treatment, and cardiac and kidney events. In this population, proximal tubule secretion capacity was not associated with any of these outcomes.

Mineralocorticoid receptor antagonists (MRAs) play a fundamental role in the treatment of heart failure (HF).^1^^,^^2^ However, MRAs are frequently underprescribed because of the risk of hyperkalemia.^3^, ^4^, ^5^ Further increasing this risk are comorbidities such as chronic kidney disease (CKD) and diabetes, which are prevalent in HF patients.^6^, ^7^, ^8^ Identifying tools to risk stratify for hyperkalemia related to MRA use among individuals with HF could help guide treatment decisions.

The kidney is the primary organ regulating potassium homeostasis. Although glomerular filtration is important for initially filtering potassium from the blood, the kidney tubules ultimately determine the final composition of potassium in the blood and urine by regulating potassium reabsorption and secretion. Biomarkers reflecting impaired kidney tubular secretion are associated with hyperkalemia risk above and beyond estimated glomerular filtration rate (eGFR) or albuminuria in non-diabetic individuals with hypertension and CKD enrolled in the Systolic Blood Pressure Intervention Trial (SPRINT).^9^^,^^10^ Reduced secretory capacity is also associated with a greater severity of interstitial fibrosis and tubular atrophy on kidney biopsy, which may provide an assessment of response to medical therapies that depend on normal kidney physiology.^11^ However, the association of secretion biomarkers with risk of hyperkalemia and cardiovascular and kidney outcomes in individuals with HF has not been evaluated.

In this analysis, we evaluated if secretion biomarkers were associated with the risk of incident hyperkalemia, response to spironolactone therapy as measured by change in Kansas City Cardiomyopathy Questionnaire (KCCQ) score and N-terminal pro-B-type natriuretic peptide (NT-proBNP) levels, and risk of cardiovascular and kidney outcomes in individuals with HF enrolled in the Treatment of Preserved Cardiac Function Heart Failure with an Aldosterone Antagonist (TOPCAT) study. We hypothesized that lower kidney secretion capacity would be associated with risk of hyperkalemia, less improvement in KCCQ scores and NT-proBNP, and greater risk of adverse kidney and cardiovascular events in individuals with HF enrolled in TOPCAT.

## Methods

### Study Population

The study design and primary results of TOPCAT have been described previously.^12^ Briefly, TOPCAT enrolled 3445 individuals ≥50 years of age with one sign or symptom of HF, a left ventricular ejection fraction ≥45%, and serum potassium <5.0 mmol/L and randomized them 1:1 to spironolactone (15 to 45 mg daily) or placebo. Key exclusion criteria included an eGFR of < 30 mL/min/1.73 m^2^ or serum creatinine ≥2.5 mg/dL. Of these 3,445 participants, a random cohort of 372 had paired blood and urine specimens with adequate sample volume at baseline stored in the National Heart, Lung and Blood Institute (NHLBI) Biologic Specimen and Data Repository (BioLINCC) available for measuring secretion biomarkers forming our analytic cohort. Of these 372 participants, 331 had paired blood specimens at baseline and 12 months for measurement of NT-proBNP. This study was approved by the institutional review board at the San Diego Veterans Affairs (E230052). Informed consent was waived because of the use of de-identified information. Data related to this manuscript is publicly available by request from the NIH Biologic Specimen and Data repository.

### Biomarker Measurements

Nine biomarkers of kidney secretion capacity (m-hydroxy Hippurate, 2-furoylglycine, tiglylglycine, cinnamoylglycine, indoxyl sulfate, 1,3,7-trimethyluric acid, 1,7-dimethyluric acid, adipic acid, and p-cresol sulfate) that are transported by organic anion transporters 1 and 3 were measured in paired blood and urine specimens at baseline. Measurements were performed using liquid chromatography-tandem mass spectrometry (LC-MS/MS) and quantified as previously described.^13^

Secretion biomarkers were compiled into a composite secretion score as in previous studies to provide a single measure of tubular secretion.^9^, ^10^, ^11^ First, the urine-to-plasma ratio (UPR) of each tubular secretion biomarker was calculated. Next, we natural log-transformed the UPR of each secretion biomarker and then standardized them to a common 0-100 scale based on the maximum and minimum level of log-transformed UPR for each individual biomarker as follows: standardized UPR = ([ln(UPR) –min(ln[UPR])] / range(ln[UPR]))∗100 where ln(UPR) represents the natural log-transformed UPR, min(ln[UPR]) represents the minimum value in the distribution of ln(UPR), and range(ln[UPR]) represents the difference between the minimum and maximum ln(UPR) values. The summary secretion score for each participant was then calculated as the average of each tubular secretion biomarkers’ standardized UPR.

NT-proBNP measurements were made using the Elecsys proBNPII reagents on the Roche Cobas e801 analyzer and performed per manufacturer specifications. All specimens were obtained from the NHLBI biorepository, shipped on dry ice and were stored frozen at −80 °C until analysis at the University of Minnesota Advanced Research and Diagnostic Laboratory.

### Outcomes

Per protocol in TOPCAT, potassium levels were measured at 4 weeks, 8 weeks, 4 months, 8 months, 12 months, and then every 6 months thereafter until the end of study. Additional measurements of potassium were performed as clinically indicated. We utilized all available potassium measurements for these outcomes.

To evaluate the association between the composite secretion score and hyperkalemia risk, we first evaluated the association between the secretion score and longitudinal potassium change defined as the absolute change between potassium measurements at subsequent time points relative to the baseline potassium measurement. Next, we evaluated the association of the secretion score with risk of incident hyperkalemia, defined as a potassium ≥5.5 mmol/L at any time during follow-up.

To evaluate the association of the composite secretion score with surrogates of efficacy of spironolactone, we evaluated the association between the secretion score and change in KCCQ score, a validated quality of life questionnaire in HF, from baseline to 12 months. We also evaluated the association of secretion score with 1) change in KCCQ ≥5 points, as this is considered a clinically significant change, and 2) change in NT-proBNP from baseline to 12 months.^14^

For cardiovascular outcomes, we evaluated the association between the composite secretion score and the primary composite cardiovascular outcome in TOPCAT, which consisted of cardiovascular death, HF hospitalization, or aborted cardiac arrest. We also evaluated the association with major adverse cardiovascular events (MACE) defined as a composite of cardiovascular death, HF hospitalization, stroke or myocardial infarction. For kidney outcomes, we evaluated the association between the secretion score and a composite of ≥40% decline in eGFR or need for renal replacement therapies.

### Statistical Analysis

Baseline characteristics were compared by randomization arm and by region (Americas vs Eastern Europe) because of previously reported discrepancies in the patient populations in these 2 regions.^15^

To assess the association of baseline secretion score with longitudinal change in potassium, we used linear mixed-effects models with random intercepts, random slopes, and an exchangeable covariance structure in unadjusted and adjusted models. In the unadjusted model, we evaluated the secretion score∗time interaction. We then adjusted for age, sex, region (Americas vs Eastern Europe), study arm, diabetes, use of angiotensin converting enzyme inhibitor (ACEi) or angiotensin receptor blocker (ARB), loop diuretic use, baseline potassium, baseline eGFR, and urine albumin-to-creatinine ratio. A sensitivity analysis was performed using only potassium values measured before study treatment (spironolactone or placebo) was discontinued, if applicable.

We used Cox proportional hazard models to evaluate the associations between baseline secretion score and the outcomes of potassium ≥5.5 mmol/L, cardiovascular outcomes, and the composite kidney outcome. Associations were assessed in unadjusted models and in models adjusted for the same variables used in the linear mixed-effects models. For the outcomes of incident hyperkalemia, a sensitivity analysis was conducted using only potassium values measured before discontinuation of study treatment (spironolactone or placebo), if applicable.

To evaluate the association between secretion score and treatment efficacy with spironolactone, we split the cohort by study arm, as an association was expected only in the treatment arm. Linear regression was used to assess the association between the secretion score and outcomes of continuous change in KCCQ and change in NT-proBNP. Logistic regression was used to evaluate the association between secretion score and change of ≥5-point improvement in KCCQ. Each outcome was first assessed in unadjusted models, followed by models adjusted for age, sex, region, diabetes, ACEi/ARB use, loop diuretic use, and eGFR.

In all analyses, effect modification by treatment arm was evaluated using the secretion score∗study arm interaction term except when study arms were assessed individually. We also evaluated for effect modification by regions of Americas (United States, Canada, Argentina, and Brazil) versus Eastern Europe (Russia and Georgia). This was performed because prior analyses reported significant differences in outcomes and treatment response between Americas and Eastern Europe with concerns that participants in Eastern Europe did not have HFpEF and did not adhere to randomized therapy.^15^ Finally, we evaluated effect modification by kidney function using the secretion score∗eGFR interaction term as prior analyses from SPRINT showed stronger associations between the secretion score and outcomes at lower eGFR values.^16^

A *P*-value of < 0.05 was considered significant for all analyses. All analyses were performed in Stata 18 (StataCorp. 2023. Stata Statistical Software).

## Results

There were multiple differences in baseline characteristics between the 3073 TOPCAT participants excluded and the 372 participants included in this analysis (Table S1). Individuals included were older, more often men, less often from Eastern Europe; had lower systolic and diastolic blood pressures; more often had histories of myocardial infarction, hypertension, atrial fibrillation, and tobacco use; had higher serum creatinine and BUN but similar eGFRs and urine albumin-to-creatinine ratio; and had lower serum sodium and potassium.

Among the 372 TOPCAT participants included in this study, the mean age was 70 ± 10 years, 45% were women, 42% were from Eastern Europe, and the mean body mass index was 32.6 ± 6.8 kg/m^2^ (Table 1). Almost all participants had hypertension (95%), 43% had atrial fibrillation, and 34% had diabetes. At baseline, the mean eGFR was 66 ± 18 mL/min/1.73 m^2^ and serum potassium was 4.2 ± 0.5 mmol/L. Baseline characteristics did not differ between the spironolactone and placebo arms. Similar to previous reports, there were multiple differences between participants enrolled in the Americas versus Eastern Europe.^15^ Participants in Eastern Europe were younger, had lower body mass index, higher systolic and diastolic blood pressures, more often had history of myocardial infarction, and less often had atrial fibrillation and diabetes mellitus, and had higher eGFRs (Table S2).

**Table 1:** Baseline Characteristics of Overall Cohort and by Treatment Arm in Subgroup of Participants From TOPCAT

Characteristics	Overall (N = 372)	Placebo (n = 193)	Spironolactone (n = 179)
Age (y), mean ± SD	70 ± 10	69 ± 10	70 ± 9
Women, n (%)	166 (45%)	85 (44%)	81 (45%)
Race, n (%)			
White	339 (91%)	172 (89%)	167 (93%)
Black	27 (7%)	17 (9%)	10 (6%)
Other	6 (2%)	4 (2%)	2 (1%)
Eastern Europe, n (%)	156 (42%)	82 (43%)	74 (41%)
BMI, kg/m^2^, mean ± SD	32.6 ± 6.8	32.2 ± 6.4	33.0 ± 7.2
SBP, mm Hg, mean ± SD	126 ± 13	125 ± 13	127 ± 13
DBP, mm Hg, mean ± SD	73 ± 10	73 ± 10	74 ± 10
Myocardial infarction, n (%)	120 (32%)	65 (34%)	55 (31%)
Hypertension, n (%)	352 (95%)	184 (95%)	168 (94%)
Diabetes mellitus, n (%)	125 (34%)	62 (32%)	63 (35%)
Atrial fibrillation, n (%)	161 (43%)	80 (42%)	81 (45%)
COPD, n (%)	47 (13%)	27 (14%)	20 (11%)
Tobacco use, n (%)	204 (55%)	106 (55%)	98 (55%)
LVEF, %, mean ± SD	60 ± 7	58 ± 8	61 ± 6
β-Blocker use, n (%)	295 (79%)	153 (79%)	142 (79%)
ACEi/ARB use, n (%)	304 (82%)	160 (83%)	144 (80%)
Diuretic use, n (%)	300 (81%)	153 (79%)	147 (82%)
Creatinine, mg/dL, mean ± SD	1.13 ± 0.31	1.11 ± 0.32	1.14 ± 0.29
eGFR, mL/min/1.73m^2^, mean ± SD	66 ± 18	68 ± 19	65 ± 18
UACR, mg/g, median [IQR]	20 (9-80)	18 (9-71)	23 (9-110)
BUN, mg/dL, mean ± SD	23 ± 12	23 ± 12	23 ± 11
Serum sodium, mmol/L, mean ± SD	140 ± 4	140 ± 4	140 ± 4
Serum potassium, mmol/L, mean ± SD	4.2 ± 0.5	4.2 ± 0.5	4.2 ± 0.4

Abbreviations: ACEi, angiotensin converting enzyme inhibitor; ARB, angiotensin receptor blocker; COPD, chronic obstructive pulmonary disease; DBP, diastolic blood pressure; eGFR, estimated glomerular filtration rate; IQR, interquartile range; SBP, systolic blood pressure; SD, standard deviation; UACR, urine albumin to creatinine ratio.

### Association of Secretion Score With Longitudinal Changes in Serum Potassium

The mean secretion score was 59 ± 12. Participants had an average of 8 measurements of potassium over a median follow-up of 3 years. Higher secretion score at baseline was not associated with a significant difference in the change in potassium levels per month during follow-up in unadjusted or adjusted analyses (Table 2). There was no significant interaction by study arm, region, or eGFR (*P* > 0.10 for all interactions). Results were similar in sensitivity analyses using only potassium measurements before discontinuation of study drug.

**Table 2:** Association of Secretion Score With Potassium Outcomes

Serial Potassium Measurements	Change in Potassium (mmol/L) Over Time Per 1-SD Higher Secretion Score	*P-*Interaction Study Arm	*P*-Interaction Region	*P*-Interaction eGFR
Unadjusted	−0.03 (−0.20 to 0.12)	0.99	0.45	0.72
Adjusted	−0.13 (−0.31 to 0.05)	0.79	0.68	0.92
**Potassium ≥5.5 mmol/L**	HR (95% CI)			
Unadjusted	0.77 (0.60-0.99)	0.50	0.69	0.32
Adjusted	0.94 (0.62-1.43)	0.64	0.10	0.34

*Note:* Adjusted model—age, sex, region (Americas vs Eastern Europe), study arm, diabetes, angiotensin converting enzyme inhibitor or angiotensin receptor blocker use, loop diuretic use, baseline potassium, estimated glomerular filtration rate (eGFR), urine albumin-to-creatinine ratio.

Abbreviation: CI, confidence interval; HR, hazard ratio.

A total of 26 individuals (7%) developed a potassium ≥5.5 mmol/L. In unadjusted analysis, a higher baseline secretion score was associated with a lower risk of hyperkalemia during follow-up (hazard ratio [HR] 0.77, 95% confidence interval [CI] 0.60, 0.99 per 1-SD higher secretion score, Table 2). However, this association was attenuated and no longer significant after adjustment for confounders (HR 0.94; 95% CI, 0.62-1.43 per 1-SD higher secretion score). There was no evidence of effect modification by study arm, region, or eGFR.

### Association of Secretion Score With KCCQ and NT-proBNP Change

Among 340 participants with KCCQ scores at baseline and 12 months, 161 were randomized to spironolactone and 179 to placebo. In the spironolactone arm, a higher secretion score was associated with a lower KCCQ score at 12 months after adjusting for confounders (change in KCCQ −3.5 per SD higher secretion score; 95% CI, −6.6 to −0.5, Table 3). No association was found in the placebo arm, and no significant interaction by region or eGFR was detected in either arm.

**Table 3:** Association of Secretion Score With Change in KCCQ

Continuous Change in KCCQ	Placebo Arm n = 179			Spironolactone Arm n = 161		
	Change in KCCQ∗ (95% CI)	*P*-Interaction Region	*P*-Interaction eGFR	Change in KCCQ∗ (95% CI)	*P*-Interaction Region	*P*-Interaction eGFR
Unadjusted	−1.3 (−4.3 to 1.7)	0.62	0.83	−2.6 (−5.2 to −0.1)	0.818	0.33
Adjusted	−1.9 (−5.2 to 1.3)	0.53	0.80	−3.5 (−6.6 to −0.4)	0.749	0.36
**≥5 point increase in KCCQ**	**OR (95% CI)**			**OR (95% CI)**		
Unadjusted	0.9 (0.7-1.3)	0.48	0.73	0.9 (0.6-1.2)	0.951	0.22
Adjusted	0.9 (0.6-1.2)	0.50	0.80	0.8 (0.5-1.2)	0.868	0.22

*Note:* Adjusted for age, sex, region (Americas vs Eastern Europe), diabetes, angiotensin converting enzyme inhibitor or angiotensin receptor blocker use, loop diuretic use, estimated glomerular filtration rate (eGFR), and urine albumin-to-creatinine ratio.

Abbreviations: KCCQ, Kansas City Cardiomyopathy Questionnaire; OR, odds ratio.

∗ Change per one standard deviation increase in secretion score.

There were 90 participants in the spironolactone arm and 79 participants in the placebo arm who had a KCCQ increase ≥5 points at 12 months. The secretion score was not associated with odds of having a ≥5 point increase in KCCQ in either study arm. No significant interaction by region or eGFR was found.

The average change in NT-proBNP was −72 pg/mL in the spironolactone arm and 196 pg/mL in the placebo arm. The secretion score was not associated with significant change in NT-proBNP in either study arm, and there was no significant interaction by region or eGFR (Table 4).

**Table 4:** Association of Secretion Score With Change in NT-proBNP

	Placebo Arm n = 149			Spironolactone Arm n = 132		
	Change in NT-proBNP∗ (95% CI)	*P*-Interaction Region	*P*-Interaction eGFR	Change in NT-proBNP∗ (95% CI)	*P*-Interaction Region	*P*-Interaction eGFR
Unadjusted	−55 (−230 to 120)	0.32	0.90	18 (−83 to 119)	0.10	0.08
Adjusted	−41 (−238 to 156)	0.62	0.64	7 (−116 to 131)	0.13	0.10

*Note:* Adjusted for age, sex, region (Americas vs Eastern Europe), diabetes, angiotensin converting enzyme inhibitor or angiotensin receptor blocker use, loop diuretic use, estimated glomerular filtration rate (eGFR), and urine albumin-to-creatinine ratio.

Abbreviations: NT-proBNP, N-terminal pro-B type natriuretic peptide.

∗ Change per one standard deviation increase in secretion score.

### Association of Secretion Score With Cardiovascular and Kidney Outcomes

A total of 76 individuals (20%) experienced the primary composite cardiovascular outcome originally defined in TOPCAT. In unadjusted analysis, a higher secretion score was associated with a lower risk of the composite outcome (HR 0.81; 95% CI, 0.66-0.99; Table 5), but this association was no longer present after adjusting for confounders (HR 1.24; 95% CI, 0.95-1.62). There was no significant interaction by study arm or region; however, a significant interaction with eGFR was observed (*P*-interaction = 0.001). To better understand this finding, we examined the association of the composite secretion score with the primary composite cardiovascular outcome across eGFR quartiles (Table S3). This exploratory analysis suggested little or no association between secretion score and cardiovascular events at lower eGFR levels, whereas in the highest eGFR quartile, higher secretion score was associated with higher risk.

**Table 5:** Association of Secretion Score With Cardiovascular and Kidney Outcomes

TOPCAT Composite Outcome	HR (95% CI)	*P*-Interaction Study Arm	*P*-Interaction Region	*P*-Interaction eGFR
Unadjusted	0.81 (0.66-0.99)	0.33	0.07	0.002
Adjusted	1.24 (0.95-1.62)	0.35	0.21	0.001
**MACE**	HR (95% CI)			
Unadjusted	0.76 (0.63-0.93)	0.56	0.02	0.006
Adjusted	1.09 (0.85-1.41)	0.82	0.05	0.003
**Composite kidney outcome**	HR (95% CI)			
Unadjusted	0.83 (0.67-1.05)	0.70	0.81	0.55
Adjusted	1.26 (0.93-1.71)	0.36	0.57	0.61

*Note:* TOPCAT composite outcome—cardiovascular death, heart failure hospitalization, or aborted cardiac arrest. MACE (major adverse cardiac events)—cardiovascular death, heart failure hospitalization, stroke, or myocardial infarction. Composite kidney outcome—≥40% decrease in estimate glomerular filtration rate or receipt of renal replacement therapy. Adjusted model—age, sex, region (Americas vs Eastern Europe), study arm, diabetes, angiotensin converting enzyme inhibitor or angiotensin receptor blocker use, loop diuretic use, baseline potassium, estimated glomerular filtration rate (eGFR), and urine albumin-to-creatinine ratio.

Abbreviations: CI, confidence interval; HR, hazard ratio.

There were 89 individuals (24%) who had our composite of MACE. Again, higher secretion score was associated with lower risk of the composite outcome in unadjusted analysis (HR 0.76; 95% CI, 0.63-0.93), but this association was not present after adjusting for confounders (HR 1.09; 95% CI, 0.85-1.41). A significant interaction by eGFR was again observed (*P*-interaction = 0.003), and exploratory analyses across eGFR quartiles showed a pattern like that observed for the primary composite cardiovascular outcome (Table S3). Additionally, a significant interaction by region was observed. In Eastern Europe, higher secretion score was associated with a higher risk of MACE in unadjusted analysis, but no association was found in the Americas (Table S4). Notably, there were only 12 events in Eastern Europe compared to 77 events in the Americas. The association between secretion score and MACE was not significant after adjustment.

There were 49 individuals who had the composite kidney outcome. The secretion score was not associated with risk of the composite kidney outcome, and there was no significant interaction by study arm, region, or eGFR (Table 5).

## Discussion

We evaluated whether biomarkers of kidney tubular secretion, summarized as a composite secretion score, were associated with risk of hyperkalemia, treatment response to spironolactone, and cardiovascular and kidney events in individuals with HFpEF enrolled in the TOPCAT study. Overall, we found that these secretion biomarkers were not associated with any of these clinical outcomes. The only significant association was between greater secretion capacity and lower KCCQ score during follow-up, though the absolute change in KCCQ score was small. These findings do not support the use of tubular secretion biomarkers to inform about future potassium changes, treatment response, or outcomes in patients with HFpEF within the limitations of the TOPCAT study cohort.

Previously, we found that each standard deviation lower secretion score was associated with 63% greater risk of ambulatory hyperkalemia in 2,089 nondiabetic individuals with hypertension and CKD in the SPRINT study.^9^ These findings prompted us to further evaluate if tubular secretion biomarkers were associated with hyperkalemia in HFpEF, a condition associated with significant risk for hyperkalemia often related to concomitant comorbidities.^17^ Although each standard deviation lower secretion score was associated with ≥0.1 mmol/L increase in potassium per month in this study in TOPCAT, this did not reach statistical significance. The secretion score was also not associated with risk of developing a potassium ≥5.5 mmol/L. Thus, we were unable to confirm an association between secretion score and risk of hyperkalemia in HFpEF patients.

Several differences in study designs and patient characteristics between SPRINT and TOPCAT may explain this lack of association. We have previously found stronger associations between the secretion score and outcomes among individuals with lower eGFRs.^16^ While the analysis from SPRINT focused exclusively on individuals with CKD, participants in TOPCAT had higher eGFRs with less CKD potentially contributing to null findings. The higher eGFR in TOPCAT also likely reflects the intentional exclusion of participants at high risk for hyperkalemia that may have biased toward individuals less likely to experience these outcomes. The frequent laboratory monitoring and protocolized MRA dose adjustments likely mitigated some of the hyperkalemia risk. Patients with HFpEF are commonly treated with ACEis, ARBs, and loop diuretics, which, despite adjusting for in our analyses, may have confounded hyperkalemia risk due to adjustment of these therapies during follow-up that was not fully captured. Finally, the well described issues with study conduct in Eastern Europe, where participants had a lower severity of HFpEF or potentially did not have HFpEF at all, may have contributed.^15^ Although we attempted to account for this by evaluating effect modification by region, there is likely residual confounding we cannot fully account for. In total, these design and population differences may have significantly contributed to the null findings observed in TOPCAT.

To exert its pharmacologic effect, spironolactone moves intracellularly from the basolateral membrane to inhibit aldosterone binding to its receptor. While spironolactone is not secreted, we had hypothesized that spironolactone’s reliance on tubular health for its mechanism of action would result in greater efficacy in individuals with greater tubular health reflected by a higher secretion score. Contrary to our hypothesis, we found a lower secretion score was associated with higher KCCQ scores in participants treated with spironolactone. A possible explanation for this finding is the previously reported association of greater interstitial fibrosis and tubular atrophy with lower secretion scores, and those individuals with greater interstitial fibrosis may derive greater benefit from MRAs.^11^ This is supported by the previously demonstrated anti-fibrotic effects of MRAs, specifically the non-steroidal MRA finerenone, which reduced CKD progression and markers of fibrosis.^17^^,^^18^ While this hypothesis is biologically plausible, the overall association for change in KCCQ was minimal, and we did not find significant associations with greater changes in KCCQ or change in NT-proBNP tempering enthusiasm for this association.

We found that a higher secretion score was associated with a lower risk of primary composite outcome from TOPCAT and MACE in unadjusted analyses; however, these associations were no longer significant after adjusting for confounders. A significant interaction with eGFR was observed for the primary composite cardiovascular outcome and MACE, suggesting that a higher secretion score was associated with a greater risk of cardiovascular events among individuals with higher eGFR values. This finding should be interpreted cautiously given the lack of a significant association in the overall adjusted analyses, the small number of events within each quartile, and the direction of the association being opposite to that hypothesized. A significant interaction by region was also observed for MACE, which paradoxically showed a greater risk of MACE with higher secretion score in Eastern Europe. Given the low event rates in Eastern Europe participants, this finding should be cautiously interpreted as well. Also, there was no association between secretion score and our composite kidney outcome. This finding differs with prior studies, which found that a lower secretion score is associated with a greater risk of CKD progression.^10^^,^^16^^,^^19^ The smaller sample size with relatively preserved kidney function here may have contributed to null findings for this outcome.

This study has several limitations in addition to those already mentioned. Our analysis may have been underpowered, as our original power calculation had been based on a sample size of 385 participants, but 13 participants were found to have inadequate sample volumes, reducing the cohort size to 372. Furthermore, given the regional differences in TOPCAT participants previously described and only 216 participants were enrolled in the Americas, this likely further reduced our power to detect significant associations. Moreover, because TOPCAT only enrolled individuals with HFpEF, our findings may not be generalizable to individuals with left ventricular ejection fraction <45%. We were only able to perform single measurement of secretion biomarkers, whereas the precision of measurements would have been improved with repeat measurements of baseline specimens. Finally, we only evaluated anion secretion biomarkers in this study, while cation secretion biomarkers may have been more informative for evaluating potassium handling by the kidney tubule.

In conclusion, in this substudy of TOPCAT, the biomarkers of tubular secretion capacity assessed in this effort are not associated with risk of hyperkalemia, treatment response, or cardiovascular and kidney outcomes in individuals with HFpEF. However, we do not believe this analysis alone precludes further evaluation of these biomarkers in HF given the limitations noted, the unique study design of TOPCAT, and the relatively small substudy of TOPCAT evaluated here.^15^^,^^20^
