# Supplementary material for: Association of Proximal Tubule Secretion With Hyperkalemia Risk, Treatment Response, and Outcomes in Heart Failure With Preserved Ejection Fraction
Source: Kidney Med. 2026 Jun 6;8(8):101428. doi: 10.1016/j.xkme.2026.101428 (PMC13396859; doi:10.1016/j.xkme.2026.101428)
Supplement: Supplementary File (PDF) — Tables S1-S4 [file mmc1.pdf]

Supplementary Material

Table S1: Comparison of Baseline characteristics in Participants in Secretion Biomarker Subcohort compared to Excluded Participants

	Included (n=372)	Excluded (n=3073)	p-value
Age, years, mean (SD)	70 (10)	68 (10)	0.03
Women, n (%)	166 (45)	1609 (52)	0.006
Race			0.32
White	339 (91)	2723 (89)	
Black	27 (7)	275 (9)	
Other	6 (2)	75 (2)	
Eastern Europe, n (%)	156 (42)	1522 (50)	0.007
Spironolactone use, n (%)	179 (48)	1543 (50)	0.48
BMI, kg/m2, mean (SD)	33 (6.8)	32 (7.1)	0.14
SBP, mmHg, mean (SD)	126 (13)	130 (14)	<0.001
DBP, mmHg, mean (SD)	73 (10)	76 (11)	<0.001
Myocardial infarction, n (%)	120 (32)	773 (25)	0.004
Hypertension, n (%)	352 (95)	2795 (91)	0.03
Diabetes mellitus, n (%)	125 (34)	993 (32)	0.67
Atrial fibrillation, n (%)	161 (43)	1053 (34)	0.001
COPD, n (%)	47 (13)	356 (12)	0.62
Tobacco use	204 (55)	1424 (46)	0.002
LVEF, %, mean (SD)	59 (7)	60 (8)	0.59
Beta-blocker use, n (%)	295 (79)	2381 (78)	0.49
ACEi/ARB use	304 (82)	2596 (85)	0.18
Diuretic use, n (%)	300 (81)	2517 (82)	0.57
Serum creatinine, mg/dL, mean (SD)	1.13 (0.31)	1.09 (0.30)	0.03
eGFR, ml/min/1.73m2, mean (SD)	66 (18)	68 (19)	0.10
UACR, mg/g, median [IQR]	20 [9, 80]	20 [7, 89]	0.30
BUN, mg/dL	23 (12)	21 (11)	0.001
Serum sodium, mmol/L, mean (SD)	140 (4)	141 (4)	<0.001
Serum potassium, mmol/L	4.2 (0.5)	4.3 (0.5)	0.004

ACEi – angiotensin converting enzyme inhibitor; ARB – angiotensin receptor blocker; COPD – chronic obstructive pulmonary disease; DBP – diastolic blood pressure; eGFR – estimated glomerular filtration rate; IQR – interquartile range; SBP – systolic blood pressure; SD – standard deviation; UACR – urine albumin to creatinine ratio

**Table S2: Baseline characteristics in Americas versus Eastern Europe in subgroup of participants from TOPCAT**			
	Americas (n=216)	Eastern Europe (n=156)	p-value
Age, years, mean (SD)	72 (10)	66 (8)	<0.001
Women, n (%)	90 (42)	76 (49)	0.21
Race			<0.001
White	183 (85)	156 (100)	
Black	27 (13)	0 (0)	
Other	6 (3)	0 (0)	
Spironolactone arm	105 (49)	74 (47)	0.91
BMI, kg/m2, mean (SD)	34.0 (7.5)	30.6 (5.1)	<0.001
SBP, mmHg, mean (SD)	125 (14)	128 (11)	0.03
DBP, mmHg, mean (SD)	70 (11)	78 (6)	<0.001
Myocardial infarction, n (%)	47 (22)	73 (47)	<0.001
Hypertension, n (%)	205 (95)	147 (94)	0.96
Diabetes mellitus, n (%)	101 (47)	24 (15)	<0.001
Atrial fibrillation, n (%)	108 (50)	53 (34)	0.003
COPD, n (%)	26 (12)	21 (14)	0.80
Tobacco use	144 (67)	60 (39)	<0.001
LVEF, %, mean (SD)	59 (7)	59 (8)	0.99
Beta-blocker use, n (%)	180 (83)	115 (74)	0.03
ACEi/ARB use	163 (76)	141 (90)	<0.001
Diuretic use, n (%)	195 (90)	105 (67)	<0.001
Serum creatinine, mg/dL, mean (SD)	1.18 (0.35)	1.05 (0.21)	<0.001
eGFR, ml/min/1.73m2, mean (SD)	64 (19)	70 (16)	0.001
UACR, mg/g, median [IQR]	20 [9, 80]	44 [13, 66]	0.37
BUN, mg/dL	25 (13)	19 (5)	<0.001
Serum sodium, mmol/L, mean (SD)	139 (3)	141 (5)	<0.001
Serum potassium, mmol/L	4.2 (0.4)	4.2 (0.5)	0.43

ACEi – angiotensin converting enzyme inhibitor; ARB – angiotensin receptor blocker; COPD – chronic obstructive pulmonary disease; DBP – diastolic blood pressure; eGFR – estimated glomerular filtration rate; IQR – interquartile range; SBP – systolic blood pressure; SD – standard deviation; UACR – urine albumin to creatinine ratio

**Table S3: Association of secretion score with cardiovascular outcomes by eGFR quartile**						
eGFR Quartile	eGFR Range (ml/min/1.73 m^2)	n	Composite Outcome		MACE	
			Events (n)	HR (95% CI)*	Events (n)	HR (95% CI)*
Q1 (lowest)	27–53	93	28	1.12 (0.82, 1.53)	30	1.01 (0.73, 1.40)
Q2	53–65	93	14	0.82 (0.38, 1.78)	17	0.69 (0.33, 1.42)
Q3	65–76	93	18	1.14 (0.61, 2.14)	21	1.01 (0.57, 1.79)
Q4 (highest)	76–176	93	16	2.57 (1.12, 4.69)	21	1.82 (1.06, 3.12)

*HR per 1 SD higher secretion score
Adjusted model - age, sex, region (Americas vs. Eastern Europe), study arm, diabetes, angiotensin converting enzyme inhibitor or angiotensin receptor blocker use, loop diuretic use, baseline potassium and estimated glomerular filtration rate (eGFR) quartile
CI – confidence interval; HR – hazard ratio

**Table S4: Association of secretion score with major adverse cardiovascular events in Eastern Europe and Americas**		
	Americas<br>HR (95% CI)	Eastern Europe<br>HR (95% CI)
Unadjusted	0.88 (0.70, 1.12)	1.94 (1.08, 3.48)
Adjusted	1.03 (0.77, 1.38)	1.63 (0.82, 3.20)
Adjusted model - age, sex, study arm, diabetes, angiotensin converting enzyme inhibitor or angiotensin receptor blocker use, loop diuretic use, baseline potassium and estimated glomerular filtration rate<br>CI – confidence interval; HR – hazard ratio		
